# Trends of paediatric hypertension screening and management in primary care before and during the coronavirus disease 2019 pandemic: A retrospective cohort study

**DOI:** 10.1093/pch/pxae079

**Published:** 2024-12-23

**Authors:** Leanne Kosowan, Rahul Chanchlani, Allison Dart, Michael Wu, Rita Costa, Alexander Singer

**Affiliations:** Department of Family Medicine, Rady Faculty of Health Sciences, University of Manitoba, Winnipeg, Manitoba, Canada; Department of Paediatrics, McMaster University, Hamilton, Ontario, Canada; Department of Paediatric and Child Health, Rady Faculty of Health Sciences, University of Manitoba, Winnipeg, Manitoba, Canada; Michael G. DeGroote School of Medicine, Faculty of Health Sciences, McMaster University, Hamilton, Ontario, Canada; Department of Family Medicine, Rady Faculty of Health Sciences, University of Manitoba, Winnipeg, Manitoba, Canada; Department of Family Medicine, Rady Faculty of Health Sciences, University of Manitoba, Winnipeg, Manitoba, Canada

**Keywords:** *Blood pressure*, *COVID-19*, *Hypertension*, *Paediatric*, *Primary health care*

## Abstract

**Objectives:**

We assessed trends in primary care paediatric blood pressure (BP) screening, follow-up, and treatment before and during the coronavirus disease 2019 (COVID-19) pandemic.

**Methods:**

Retrospective cohort study using electronic medical records from the Canadian Primary Care Sentinel Surveillance Network to capture paediatric visits (aged 3 to 18) between January 1, 2011, and December 31, 2020. Time-series analysis was performed using documentation of monthly BP, high BP, follow-up of abnormal BP, and antihypertensive prescribing. We assessed differences between pre (January 1, 2011 to March 11, 2020) and during COVID-19 (March 12, 2020 to December 31, 2020).

**Results:**

Of 343,191 paediatric patients, 30.9% had ≥1 paediatric BP documented. Documentation of BP increased each year from 17.3% in 2011 to 19.8% in 2019 (β = 0.05, 95% CI 0.04, 0.07, P < 0.001), with a decrease in trend in 2020 to 11.0% (β = −16.95, 95% CI −18.91, −14.99, P < 0.001). There was an increasing pre-pandemic trend for laboratory screening and prescribing (β = 0.12, 95% CI 0.1, 0.14, P < 0.0001; β = 0.02, 95% CI 0.02, 0.02, P < 0.0001). During the COVID-19 pandemic, laboratory screening further increased (24.5% to 31.1%; β = 5.19, 95% CI 2.03, 8.35, P = 0.002), whereas there was no significant change in prescribing trends (1.3% to 1.4%; β = 0.15, 95% CI −0.01, 0.32, P = 0.07).

**Conclusions:**

Documentation of BP increased annually, then declined precipitously during the COVID-19 pandemic. Despite lower BP screening and follow-up, the prevalence of hypertension and antihypertensive prescribing remained stable. Clinical practice trends in primary care highlight areas to improve the care and management of hypertensive paediatric patients.

## Graphical Abstract

**Graphical Abstract:**
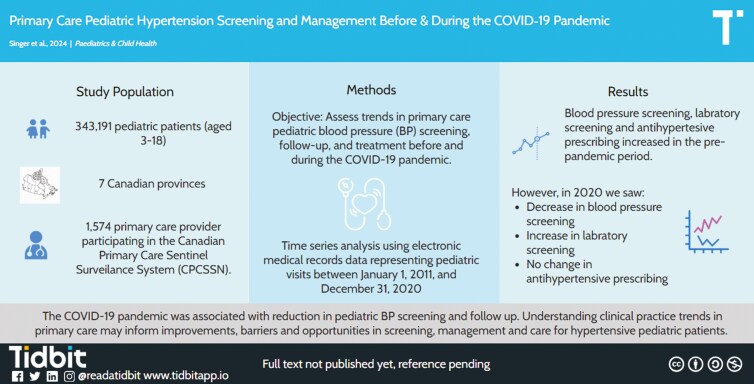

Despite its relatively high prevalence (~5%), paediatric hypertension is often underrecognized and inadequately treated (1–8). Paediatric hypertension is associated with target organ damage and adverse cardiovascular outcomes later in life (1,2,9–16). The 2016 Canadian (17) and 2017 American (18) paediatric hypertension guidelines provided comprehensive recommendations specific to blood pressure (BP) screening and management (17,18). Although current paediatric hypertension guidelines suggest that BPs should be measured at least annually in children ≥3 years of age (17,18), research suggests that between 40% and 60% of encounters do not assess paediatric BPs (1,6–8,19). Pediatric BP screening is associated with patient characteristics such as sex, obesity, and comorbidities (1,5–7).

Resulting from the Sever acute respiratory syndrom coronavirus 2 (SARS-CoV-2) coronavirus disease 2019 [COVID-19], there were significant changes in clinical care practices to accommodate public health recommendations and reduce the spread of the virus including social distancing and rapid adoption of virtual healthcare services (20). Several studies have observed the health impacts of the COVID-19 pandemic including direct and indirect effects on disease prevention, recognition, and management (19–25). Implications of changes in care provision to accommodate public health recommendations, particularly around paediatric BP screening, are not well described. In several other domains of prevention and screening, indicators have notably decreased, for example, cancer screening (25–28) and paediatric immunization (29). Cunningham et al. (30) reported a decrease in BP screening among US health departments due to the COVID-19 pandemic response, with 67.2% of providers indicating a reduction in BP screening. Given already lower rates of paediatric BP screening globally, this study assessed the impact of changes in care patterns related to the COVID-19 pandemic response on paediatric BP screening, follow-up, and treatment in primary care settings.

## METHODS

This retrospective cohort study used de-identified electronic medical record (EMR) data from 1574 primary care family physicians, nurse practitioners, and community paediatricians participating in the Canadian Primary Care Sentinel Surveillance Network (CPCSSN). CPCSSN generates a pan-Canadian EMR repository representing primary care providers located in seven Canadian provinces (BC, AB, MB, ON, QC, NS, and NL) with care provision to >1,800,000 Canadians. This study accessed Anatomical Therapeutic Chemical (ATC) Classification codes representing medication prescribing (31), International Classification of Disease, ninth edition, clinical modification (ICD-9-CM) codes representing medical diagnoses, and Logical Observation Identifiers Names and Codes representing laboratory ordering.

### Study population

Our study included patients aged 3 to 18 years with ≥1 encounter with a primary care provider between January 1, 2011, and December 31, 2020. We focused on this period to assess the impact of the COVID-19 pandemic on screening in Canadian primary care settings (19). We excluded encounters with children <3 years of age as routine BP screening is not recommended by existing guidelines (16,17). Children could age in or out of our study cohort each year. We also excluded children with chronic kidney disease defined based on eGFR <90 mL/min/1.73 m^2^ based on the CKiD estimated glomerular filtration rate (eGFR) equation (1,6,32), which may influence BP screening and follow-up.

### Outcomes

Annual paediatric BP screening was defined as the proportion of the population aged 3 to 18 with BP documentation and ≥1 visit to a primary care provider the same year. We assessed the impact of BP screening trends on hypertension by capturing trends in high BP and hypertension. High BP was defined as a combined outcome of elevated, stage 1 or stage 2 hypertension using the AAP 2017 guidelines: BP ≥90th percentile for age, sex, and height [3 to 12 years old] or ≥120/80 mmHg [≥13 years old] (18). Patients with two consecutive high BPs documented were identified as having paediatric hypertension. Further, we assessed changes in care provisions to those captured with high BP. Although guidelines recommend BP follow-up within 2 weeks, we assessed for a second BP documented within 6 months, as a practical application. Follow-up BP that occurred during the pandemic may follow a high BP during the pre-pandemic period. We assessed screening laboratory investigations within 2 months of a high BP: creatinine, thyroid profile, electrolytes, Hemoglogin A1c (HbA1c), aspartate transaminase (AST)/alanine transaminase (ALT), and lipid profile. We assessed antihypertensive medication prescriptions including (ATC: C02), ACE inhibitors (ATC: C09), beta blocker (ATC: C07), Diuretic (ATC: C03AA03, C03BA04, C03BA08, C03BA11, C03DB01, C03DB02, C03EA01), and calcium channel blockers (ATC: C08CA01, C08CA02, C08DA01) (31).

### Variables

Patient covariates included age at first encounter, age at high BP, sex, urban vs. rural residence (derived from patient’s postal code), social and material deprivation index, body mass index (BMI), and diagnosis of diabetes mellitus. The social and material deprivation index is a marker of socioeconomic status that uses a postal code conversion file to link a patient’s six-character postal code to quintiles from least deprived (score of 1) to most deprived (score of 5) (33,34). Material deprivation is represented by neighbourhood rates of high school diploma attainment, employment-population ratio, and average personal income. Social deprivation refers to the proportion of persons living alone, individuals separated, divorced or widowed, and single-parent families (32,33). Social and material deprivation index was not available for 8% (n = 26,517) of patients in the study. Children were considered overweight based on Centers for Disease Control and Prevention (CDC) guidelines; if their BMI was >97%tile (if age ≤5 years) or >85%tile (if age >5 years) (35). CPCSSN case definition was used to flag patients with diabetes (36). At a clinic level, we assessed if the patient visited a rural or urban clinic using the first three characters of the clinic’s postal code.

### Analysis

We created annual cohorts of active patients for each study year and assessed the frequency and proportion of each outcome of interest. Patient characteristics were described using mean, standard deviation (SD), frequency, and percentage (%). We assessed differences in outcomes of interest using chi-square and t-test. We assessed differences between patients pre (January 1, 2011, to March 11, 2020) and during the (March 12, 2020, to December 31, 2020) COVID-19 pandemic for each of the outcomes of interest using a generalized estimate equations model.

Interrupted time-series analysis examines the impact of the COVID-19 pandemic on BP screening, BP follow-up, laboratory follow-up, and antihypertensive prescribing (37,38). Models included a time variable for overall study months (β0, January 1, 2011, to December 31, 2020) and variables representing pre- and during the pandemic (January 1, 2011, to March 11, 2020; March 12, 2020, to December 31, 2020). Dichotomous time-period variables provided an effect estimate of the level change (β2, immediate effect) at the start of the pandemic while controlling for the pre-pandemic trend (β1). Further, our model captured a change in trend during the pandemic (β3, sustained effect). In 2016 (17) and 2017 (18) revisions to the paediatric hypertension recommendations provided updated guidance on BP screening and management, we, therefore, performed a sensitivity analysis to assess the impact of the pandemic on outcomes of interest when the pre-pandemic period was January 1, 2017, to March 11, 2020. We tested for autocorrelation using the Durbin–Watson statistic. Statistical analyses were conducted using SAS V9.4 (SAS Institute Inc., Cary, NC). This study was approved by the Health Research Ethics Board at the University of Manitoba. Informed consent was obtained from primary care providers. Consent was not obtained from patients as the data were de-identified during extraction from the EMR.

## RESULTS

There were 343,309 paediatric patients (aged 3 to 18 years) that had ≥1 visit with a primary care provider between January 1, 2011, and December 31, 2020. Approximately 40.0% of paediatric patients had an in-person or virtual visit in 2018 (38.7%), 2019 (41.9%), and 2020 (37.9%). Among these patients, 30.9% (n = 105,948) had at least one paediatric BP screening documented in the EMR (Supplementary Table 1).

### BP screening

**Figure 1.**
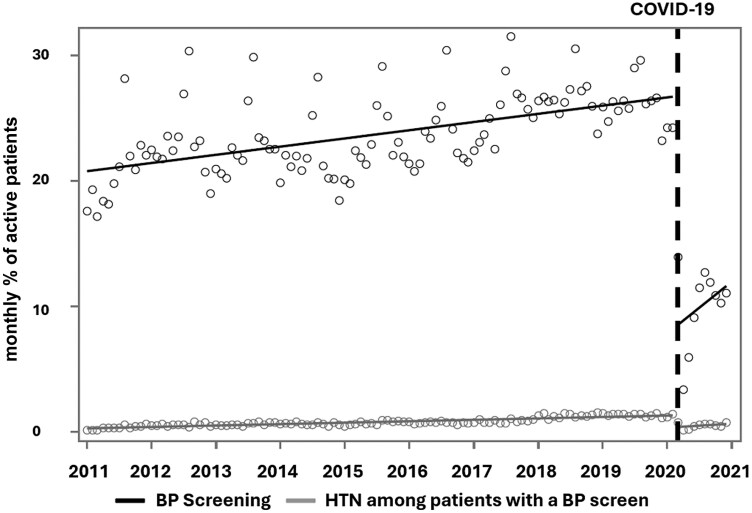
Trends in paediatric blood pressure screening and hypertension among paediatric patients in the Canadian Primary Care Sentinel Surveillance Network between January 1, 2011, and December 31, 2020

Documentation of BP increased each year from 17.3% (2011) to 19.8% (2019), however, significantly decreased to 11.0% in 2020 (P < 0.0001) (Figure 1). Patients with a BP screen in 2020 were significantly more likely to be female (54.8% vs. 53.5%, P = 0.002) and visit an urban clinic (95.6% vs. 94.8%, P = 0.001) compared to patients with a BP screen (2011 to 2019) (Table 1). Table 2 presents the time-series model showing an increasing trend pre-COVID (β = 0.05, 95% CI −0.04, 0.07, P < 0.001) followed by an immediate reduction in monthly BP screening in March 2020 (19.8% to 11.0%, β = −16.95, 95% CI −18.91, −14.99, P < 0.001). On sensitivity analysis, there continued to be a significant decrease (β = −16.59, 95% CI −18.92, −14.27, P < 0.001) (Supplementary Table 2).

**Table 1.** Characteristics of paediatric patients with a BP screen that saw a primary care provider pre- and during the COVID-19 pandemic

	Pre-COVID-19	During COVID-19	P-value
Blood pressure (BP) screening			
Female (vs. male) patient, n (%)	53.5% (53,976/100,989)	54.8% (7861/14,334)	**0.002**
Age at BP screen, mean (SD)	11.6 (5.1)	9.9 (4.8)	**<.0001**
Urban (vs. rural) residency, n (%)	84.5% (83,804/99,192)	85.0% (12,039/14,140)	0.099
Maternal and social deprivation			
1 (least deprived)	20.4% (19,695/96,414)	19.2% (2604/13,557)	0.285
2	21.0% (20,242/96,414)	19.5% (2646/13,557)	
3	21.6% (20,808/96,414)	23.6% (3205/13,557)	
4	18.6% (17,884/97,414)	17.6% (2385/13,557)	
5 (most deprived)	18.5% (17,785/96,414)	20.0% (2717/13,557)	
Overweight or obese, n (%)	35.7% (30,332/84,980)	38.9% (5086/13,067)	<.0001
Diabetes, n (%)	0.9% (913/101,013)	1.0% (139/14,353)	0.43
Urban (vs. rural) clinic, n (%)	94.8% (95,753/101,013)	95.6% (13,714/14,353)	**0.001**
Antihypertensive medication, n (%)	2.5% (2472/101,013)	4.8% (689/14,353)	**<.0001**
Patients with two consecutive high BP			
Female (vs. male) patient, n (%)	51.0% (4237/8306)	53.0% (465/878)	0.272
Age at second high BP, mean (SD)	14.2 (4.1)	14.0 (4.3)	0.243
Urban (vs. rural) residency, n (%)	83.4% (6826/8184)	82.6% (715/866)	0.527
Maternal and social deprivation			
1 (least deprived)	17.5% (1397/8007)	12.8% (109/850)	**0.004**
2	19.9% (1592/8007)	18.4% (156/850)	
3	21.0% (1685/8007)	23.3% (198/850)	
4	17.6% (1406/8007)	18.5% (157/850)	
5 (most deprived)	24.1% (1927/8007)	27.1% (230/850)	
Overweight or obese, n (%)	59.7% (4431/7420)	54.2% (429/792)	**0.003**
Diabetes, n (%)	2.6% (215/8306)	2.1% (18/878)	0.335
Urban (vs. rural) clinic, n (%)	94.3% (7836/8306)	93.2% (818/878)	0.156
Antihypertensive medication, n (%)	5.6% (470/8306)	4.1% 36/878	0.069

BP, blood pressure; COVID-19, coronavirus disease 2019; SD, standard deviation Bold text indicates significance at α <0.05

**Table 2.** Results of interrupted time-series analysis to show the impact of COVID-19 social distance measures on BP screening and hypertension prevalence

	Beta coefficient (95% CI)	P-value
Monthly blood pressure screening		
Pre-COVID trend (2011–2019) (β1)	0.05 (0.04, 0.07)	**<0.0001**
COVID-19 impact (March 2020) (β2)	−16.95 (−18.91, −14.99)	**<0.0001**
COVID-19 trend (2020) (β3)	0.34 (−0.47, 1.16)	0.3569
DW 1.034, ACF 0.477		
Monthly prevalence of hypertension (all paediatric patients)		
Pre-COVID trend (2011–2019) (β1)	0.01 (0.008, 0.01)	**<0.0001**
COVID-19 impact (March 2020) (β2)	−0.86 (−0.98, −0.73)	**<0.0001**
COVID-19 trend (2020) (β3)	0.03 (−0.02, 0.08)	0.2649
DW 1.097, ACF 0.442		
Monthly prevalence of hypertension (patients with a BP screen)		
Pre-COVID trend (2011–2019) (β1)	0.03 (0.03, 0.04)	**<0.0001**
COVID-19 impact (March 2020) (β2)	−0.33 (−0.82, 0.15)	0.1778
COVID-19 trend (2020) (β3)	0.12 (−0.07, 0.31)	0.1793
DW 1.24, ACF 0.345		

ACF, 1st order autocorrelation; BP, blood pressure; 95% CI, 95% confidence interval; COVID-19, coronavirus disease 2019; DW, Durbin–Watson D Bold text indicates significance at α <0.05

### High BP

Among patients with ≥1 BP, 40.2% (n = 35,591) had ≥1 high BP reading, and 9184 patients with two consecutive high BP readings indicating 8.7% of patients with a BP documented (2.7% of the study cohort) had hypertension.

Among all paediatric patients hypertension significantly increased each year from 0.3% in 2011 to 1.3% in 2019 (β = 0.01, 95% CI 0.01, 0.01, P < 0.001), followed by a significant decline to 0.7% in 2020 (β = −0.86, 95% CI −0.98, −0.73, P < 0.001) (Table 2). Among patients with a BP screen, we saw an increasing trend of hypertension pre-COVID (1.6% to 8.2%, β = 0.03, 95% CI 0.03, 0.04, P < 0.001), with no significant change in hypertension in March 2020 (8.2% to 7.9%; β = −0.33, 95% CI −0.82, 0.15, P = 0.178) (Table 2, Figure 1). Patients with hypertension in 2020 were more likely to reside in an area with more deprivation (27.1% vs. 24.1%, P = 0.004) and were less likely to be overweight or obese (54.2% vs. 59.7%, P = 0.003) compared to pre-COVID (Table 1). On sensitivity analysis, there was an immediate decrease in hypertension in March 2020 (β = −1.07, 95% CI −1.26, −0.89, P = 0.006) (Supplementary Table 2).

### Follow-up BP screening

**Figure 2.**
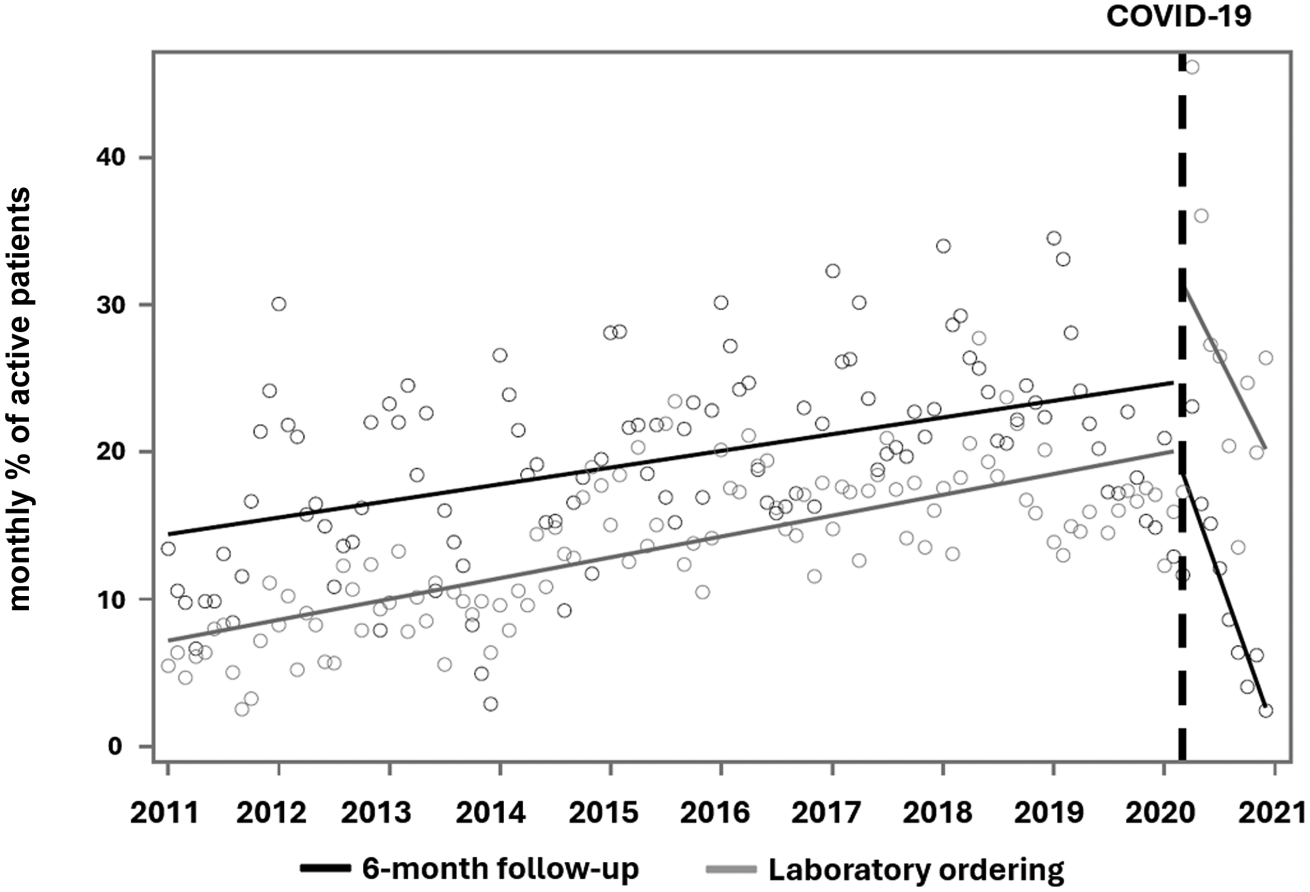
Paediatric patients with elevated blood pressure that had a 6-month follow-up and screening labs for secondary causes of hypertension between January 1, 2011, and December 31, 2020

There were 35,591 patients with ≥1 high BP between 2011 and 2020. Among those, 44.1% (n = 15,700) had a follow-up BP within 6 months. Between 2011 and 2019, BP follow-up increased (13.2% to 23.5%, β = 0.1, 95% CI 0.07, 0.14, P < 0.001), followed by a reduction in 2020 (23.5% to 16.3%; β = −14.52, 95% CI −18.83, −10.21, P < 0.001) (Table 3, Figure 2). On sensitivity analysis, we continued to observe reductions (Supplementary Table 2).

**Table 3.** Results of interrupted time-series analysis to show the impact of COVID-19 social distance measures on abnormal BP follow-up and medication prescriptions

	Beta coefficient (95% CI)	P-value
6-month blood pressure follow-up		
Pre-COVID trend (2011–2019) (β1)	0.09 (0.06, 0.13)	**<0.0001**
COVID-19 impact (March 2020) (β2)	−14.52 (−18.83, −10.21)	**<0.0001**
COVID-19 trend (2020) (β3)	−1.78 (−2.7, −0.86)	**0.0021**
DW 0.903, ACF 0.539		
Laboratory follow-up of hypertension		
Pre-COVID trend (2011–2019) (β1)	0.12 (0.1, 0.14)	**<0.0001**
COVID-19 impact (March 2020) (β2)	5.19 (2.03, 8.35)	**0.0015**
COVID-19 trend (2020) (β3)	−1.25 (−3.6, 1.1)	0.2542
DW 1.152, ACF 0.416		
Prescribing of medication to paediatric hypertension patients		
Pre-COVID trend (2011–2019) (β1)	0.02 (0.02, 0.02)	**<0.0001**
COVID-19 impact (March 2020) (β2)	0.15 (−0.01, 0.32)	0.07
COVID-19 trend (2020) (β3)	−0.04 (−0.14, 0.06)	0.3669
DW 1.169, ACF 0.412		

ACF, 1st order autocorrelation; BP, blood pressure; 95% CI, 95% confidence interval; COVID-19, coronavirus disease 2019; DW, Durbin–Watson D Bold text indicates significance at α <0.05

### Laboratory follow-up

Among 35,591 patients with ≥1 high BP, (8.2%) 2914 had subsequent screening laboratory tests. There was a significant increase in laboratory screening between 2011 and 2019 (8.8% to 24.5%; β = 0.12, 95% CI 0.1, 0.14, P < 0.0001), with a further increase to 31.1% in March 2020 (β = 5.19, 95% CI 2.03, 8.35, P = 0.02) (Table 3, Figure 2). Patients with laboratory screening in 2020 were older [14.9 (3.3) vs. 13.9 (3.3) years, P = 0.0001], and more likely to reside in an area with high deprivation (27.3% vs. 23.1%, P = 0.024) compared to patients with laboratory screening 2011 to 2019 (Table 4). On sensitivity analysis, we continued to observe a significant increase in March 2020 (β = 9.88, 95% CI 4.73, 15.04, P = 0.0004) (Supplementary Table 2).

**Table 4.** Characteristics of paediatric patients with a follow-up for a high BP screen pre- and during the COVID-19 pandemic

	Pre-COVID-19	During COVID-19	P-value
BP follow-up			
Female (vs. male) patient, n (%)	58.8% (7396/12,589)	56.5% (886/1569)	0.83
Age at follow-up, mean (SD)	13.3 (4.1)	13.4 (4.1)	0.352
Urban (vs. rural) residency, n (%)	85.0% (10,550/12,419)	84.7% (1311/1547)	0.623
Maternal and social deprivation			
1 (least deprived)	18.3% (2203/12,023)	14.1% (210/1485)	0.645
2	19.9% (2387/12,023)	17.8% (264/1485)	
3	22.2% (2674/12,023)	23.5% (349/1485)	
4	18.2% (2183/12,023)	19.8% (294/1485)	
5 (most deprived)	21.4% (2576/12,023)	24.8% (368/1485)	
Overweight or obese, n (%)	44.5% (4935/11,096)	47.3% (683/1444)	0.338
Diabetes, n (%)	1.5% (194/12,601)	1.7% (26/1572)	0.97
Urban (vs. rural) clinic, n (%)	95.6% (12,050/12,601)	95.7% (1504/1572)	0.806
Antihypertensive medication, n (%)	6.9% (872/12,601)	11.6% (183/1572)	0.238
Laboratory follow-up			
Female (vs. male) patient, n (%)	55.1% (1327/2407)	53.7% (380/708)	0.49
Age at laboratory result, mean (SD)	13.9 (3.3)	14.9 (3.3)	**0.0001**
Urban (vs. rural) residency, n (%)	83.3% (1985/2384)	82.0% (574/700)	0.435
Maternal and social deprivation			
1 (least deprived)	17.1% (398/2334)	14.9% (102/684)	**0.024**
2	19.1% (445/2334)	18.4% (126/684)	
3	22.6% (528/2334)	21.4% (146/684)	
4	18.1% (423/2334)	18.0% (123/684)	
5 (most deprived)	23.1% (540/2334)	27.3% (187/684)	
Overweight or obese, n (%)	65.5% (1371/2093)	64.2% (408/636)	0.529
Diabetes, n (%)	5.3% (128/2407)	6.5% (46/708)	0.23
Urban (vs. rural) clinic, n (%)	92.3% (2221/2407)	92.7% (656/708)	0.372
Antihypertensive medication, n (%)	8.0% (193/2407)	7.5% (53/708)	**0.02**

BP, blood pressure; COVID-19, coronavirus disease 2019; SD, standard deviation Bold text indicates significance at α <0.05

### Medication prescribing

**Figure 3.**
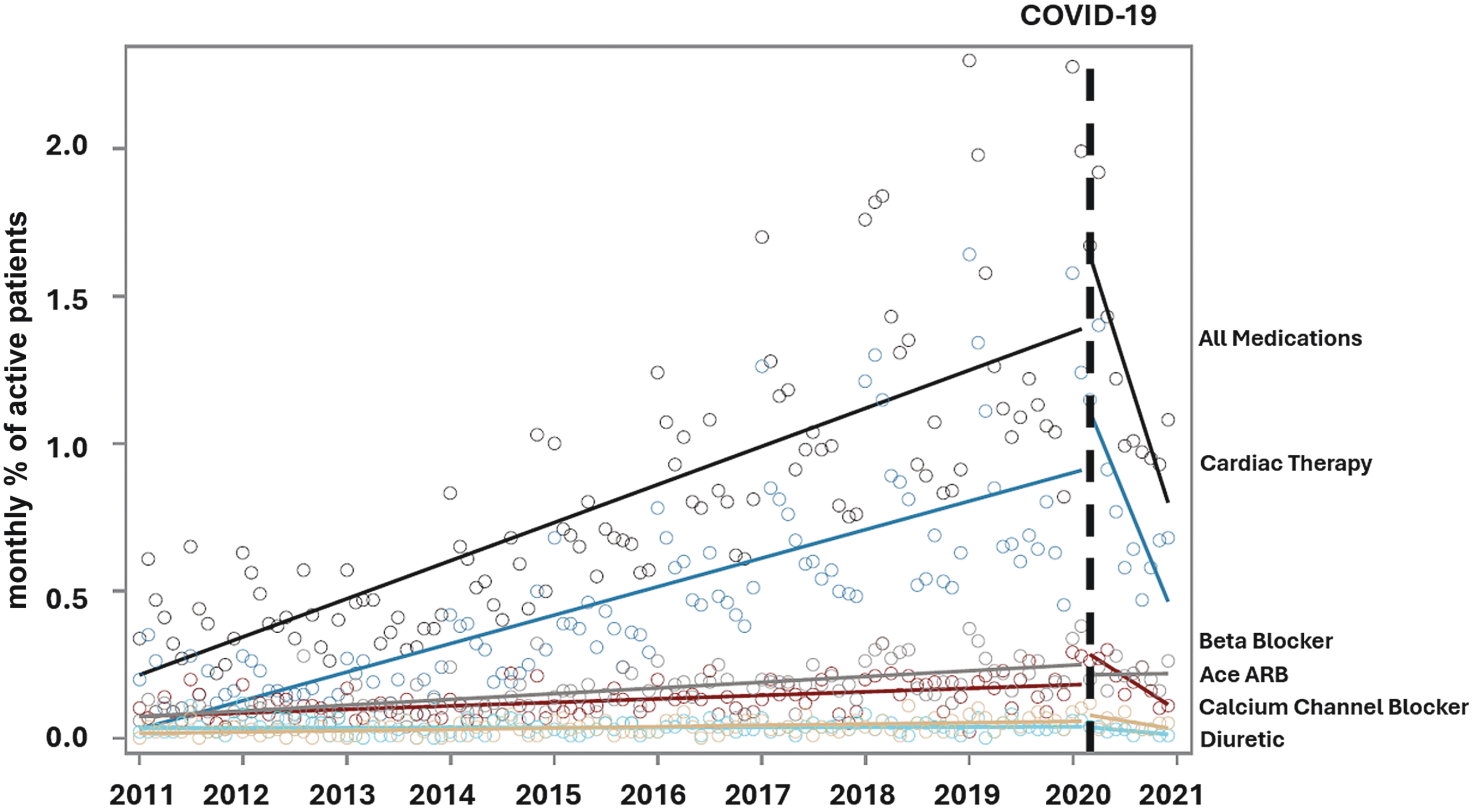
Trends in antihypertensive prescribing among paediatric patients with a visit to a primary care provider participating in the Canadian Primary Care Sentinel Surveillance Network between January 1, 2011, and December 31, 2020

There were 10,094 paediatric patients (2.9%) prescribed antihypertensive medication between 2011 and 2020. Male patients were more likely to be prescribed antihypertensive medication (66.3% vs. 33.7%, P < 0.001) compared to female patients, and 40.8% of patients prescribed medication were overweight or obese. Incident prescriptions for antihypertensive medication increased between 2011 and 2019 (0.4% to 1.3%; β = 0.02, 95% CI 0.02, 0.02, P < 0.001), with increases largely attributed to antihypertensive medications (C02) (0.2% to 0.9%) (Figure 3, Table 3). There was no significant change in prescribing in March 2020 (1.3% to 1.4%; β = 0.15, 95% CI −0.01, 0.32, P = 0.07) (Table 3, Figure 3). There were no observed differences in patients prescribed antihypertensive medication in 2020 compared with 2011 to 2019 (Table 5).

**Table 5.** Characteristics of paediatric patients with antihypertensive medication prescribed pre- and during the COVID-19 pandemic

	Pre-COVID-19	During COVID-19	P-value
Female (vs. male) patient, n (%)	35.4% (959/2707)	35.0% (461/1319)	0.764
Age at prescription, mean (SD)	11.4 (3.2)	11.6 (3.1)	0.115
Urban (vs. rural) residency, n (%)	82.7% (2200/2661)	81.5% (1049/1287)	0.368
Maternal and social deprivation			
1 (least deprived)	17.5% (443/2532)	16.9% (207/1223)	0.424
2	21.8% (553/2532)	20.2% (247/1223)	
3	22.7% (575/2532)	22.7% (277/1223)	
4	17.1% (433/2532)	18.2% (223/1223)	
5 (most deprived)	20.9% (528/2532)	22.0% (269/1223)	
Overweight or obese, n (%)	41.3% (980/2373)	40.6% (483/1190)	0.674
Diabetes, n (%)	1.8% (49/2707)	1.7% (23/1319)	0.881
Urban (vs. rural) clinic, n (%)	92.9% (2516/2707)	92.1% (1215/1319)	0.342

BP, blood pressure; COVID-19, coronavirus disease 2019; SD, standard deviation

## DISCUSSION

This study describes the impact of the COVID-19 pandemic on paediatric primary care. Immediately following the initiation of pandemic restrictions, we observed a 44% reduction in documented paediatric BP screening and a 14% reduction in follow-up BP contributing to decreases in documented paediatric hypertension. Interestingly, we did not observe a reduction in laboratory follow-up or antihypertensive prescribing.

Similar to this study, Ding et al. (1) reported an increasing trend in BP documentation between 2011 and 2017. The COVID-19 pandemic response led to considerable direct and indirect impacts on disease prevention, recognition, and management (21,23,25). Our findings are consistent with other literature demonstrating reductions in screening (11–25,30). Cunningham et al. (30) surveyed US health departments reporting a 67.2% reduction in BP screening speculating association with staff reassignment. We reported a lower reduction in screening compared to other literature (25,30). Providers caring for paediatric patients may have been less likely to be reassigned during the pandemic, or there may have been less delay and avoidance of care within this population. Similar rates in paediatric hypertension prior to and during the COVID-19 pandemic suggest that patients at heightened risk for paediatric hypertension were still screened.

Our findings re-enforce Ruth et al. (20) assertion, that without significant increases in resources missed opportunities for disease screening during the COVID-19 pandemic will not be captured. Patient characteristics may influence BP documentation (1,27,33). We report no change in BP documentation associated with obesity, or comorbidities, however, did find an increase in documentation among female patients and patients who attend an urban clinic. Although it was anticipated that differences in BP screening by socioeconomic status (SES) could have been exacerbated due to pandemic-related social distancing practices (34,39), we did not find a significant difference in BP documentation based on deprivation index. Despite this finding, it is crucial to recognize that rates of BP screening and BP follow-up did decrease following COVID-19 disrupting the previously noted increasing trend of BP documentation (1,17,18,34,39). Delayed or missed preventative screening combined with health behaviour changes may compound the effect on health outcomes and uncontrolled hypertension persisting beyond the pandemic (25,30).

Despite decreases in BP documentation, there was not a similar decrease in antihypertensive prescribing. This finding is different from other literature suggesting a decrease in medication therapy due to the COVID-19 pandemic (25). This may imply that primary care providers still managed paediatric hypertension throughout 2020, likely by leveraging virtual care. Although virtual care can support population-level risk reduction through the promotion of routine paediatric BP screening, follow-up and monitoring impacts of the COVID-19 pandemic demonstrate the need to continue, relaunch, or start new BP screening programs as a chronic disease prevention strategy (6,20,21,30,39–42).

### Limitations

Despite assessing paediatric BP documentation from a large pan-Canadian representative dataset (43), we cannot be certain our cohort is representative of other jurisdictions. This analysis focused on data up to January 2021 to parallel literature aimed at informing prevention activities during national emergencies. Our dataset did not include more recent data so cannot describe trends from January 2021 to the present. Similarly, due to less data points during COVID-19, the significance of β3 should be interpreted with caution. This retrospective study sought associations between BP screening and the COVID-19 pandemic, future research should explore associations between BP screening and comorbidities. Due to the nature of this study, we are unable to confirm causation as there may be other factors not captured, including access to emergency departments or specialty clinics. Further, we relied on primary care provider documentation in EMR, which could overestimate or underestimate prevalence due to missing diagnoses, and incomplete documentation. Documentation of urine analysis and renal ultrasound are less consistent in primary care EMR data. Importantly, BPs documented outside of suggested fields may be of less clinical utility as it may be harder to locate at a future appointment. Further, the US Preventative Task Force statement suggests insufficient evidence to support paediatric BP screening, which may influence BP screening rates despite other guidelines recommending the practice (44,45).

## CONCLUSIONS

The COVID-19 pandemic was associated with a reduction in paediatric BP screening and follow-up. Understanding primary care clinical practice trends informs improvements in screening, management, and care for hypertensive paediatric patients as well as highlights barriers and opportunities to inform future national emergencies. Research should monitor BP screening and follow-up as new care strategies are developed and implemented.

## Supplementary Material

pxae079_suppl_Supplementary_Table_S1

pxae079_suppl_Supplementary_Table_S2
