# Supplementary material for: Trends of paediatric hypertension screening and management in primary care before and during the coronavirus disease 2019 pandemic: A retrospective cohort study
Source: Paediatr Child Health. 2024 Dec 23;30(3):140–9. doi: 10.1093/pch/pxae079 (PMC12208368; doi:10.1093/pch/pxae079)
Supplement: pxae079_suppl_Supplementary_Table_S1 [file pxae079_suppl_supplementary_table_s1.docx]

| Supplementary Table 1: Pediatric blood pressure (BP) screening between January 1, 2011 and December 31, 2020 among patients that saw a CPCSSN participating provider | | | |
| --- | --- | --- | --- |
|  | Patients without a pediatric BP screening  n=237,361 | Patients with a pediatric BP screening  n=105,948 | p-value |
| Female (vs male) patient, n (%) | 116,653 (49.2) | 56,637 (53.5) | <.0001 |
| Age at first encounter, mean (SD) | 6.1 (4.4) | 8.0 (4.6) | <.0001 |
| Urban (vs rural) residency, n (%) | 187,874 (82.1) | 87,775 (84.4) | <.0001 |
| Maternal and social deprivation | | | |
| 1 (least deprived) | 42,656 (19.8) | 20,456 (20.3) | <.0001 |
| 2 | 51,700 (24.0) | 21,118 (20.9) |  |
| 3 | 47,080 (21.8) | 22,015 (21.8) |  |
| 4 | 39,017 (18.1) | 18,751 (18.6) |  |
| 5 (most deprived) | 35,247 (16.3) | 18,634 (18.5) |  |
| Overweight or obese, n (%) | 32,251 (25.3) | 30,332 (35.7) | <.0001 |
| Diabetes, n (%) | 1,349 (0.6) | 955 (0.9) | <.0001 |
| Antihypertensive medication, n (%) | 2,373 (1.0) | 2,569 (2.4) | <.0001 |
| **Abbreviations**  BP: blood pressure  CPCSSN: Canadian Primary Care Sentinel Surveillance Network  SD: standard deviation | | | |
