# Supplementary material for: Trends of paediatric hypertension screening and management in primary care before and during the coronavirus disease 2019 pandemic: A retrospective cohort study
Source: Paediatr Child Health. 2024 Dec 23;30(3):140–9. doi: 10.1093/pch/pxae079 (PMC12208368; doi:10.1093/pch/pxae079)
Supplement: pxae079_suppl_Supplementary_Table_S2 [file pxae079_suppl_supplementary_table_s2.docx]

**Supplementary Table 2: Results of sensitivity analysis showing interrupted time-series analysis of the impact of COVID-19 on BP screening, abnormal BP follow up, hypertension prevalence and laboratory and medication prescriptions between January 1, 2017 to December 31, 2020.**

|  | Beta Coefficient (95% CI) | | P-value |
| --- | --- | --- | --- |
| **Monthly blood pressure screening** | | | |
| Pre-COVID trend (2011-2019) (β1) | 0.54 (-0.04, 0.08) | | 0.5399 |
| COVID-19 impact (March 2020) (β2) | -16.59 (-18.92, -14.27) | | <.0001 |
| COVID-19 trend (2020) (β3) | 0.34 (-0.47, 1.16) | | 0.3569 |
| DW 1.316, ACF 0.319 | | | |
| **Monthly prevalence of hypertension (all paediatric patients)** | | | |
| Pre-COVID trend (2011-2019) (β1) | 0.02 (0.01, 0.02) | | <.0001 |
| COVID-19 impact (March 2020) (β2) | -1.07 (-1.26, -0.89) | | <.0001 |
| COVID-19 trend (2020) (β3) | 0.03 (-0.02, 0.08) | | 0.2649 |
| DW 1.475, ACF 0.251 | | | |
| **Monthly prevalence of hypertension (patients with a BP Screen)** | | | |
| Pre-COVID trend (2011-2019) (β1) | 0.06 (0.04, 0.08) | <.0001 | |
| COVID-19 impact (March 2020) (β2) | -1.14 (-1.86, 0.43) | 0.0023 | |
| COVID-19 trend (2020) (β3) | 0.12 (-0.07, 0.31) | 0.1793 | |
| DW 1.343, ACF 0.280 | | | |
| **6-month blood pressure follow-up** | | | |
| Pre-COVID trend (2011-2019) (β1) | -0.2 (-0.25, -0.06) | | 0.0077 |
| COVID-19 impact (March 2020) (β2) | -7.23 (-12.31, -2.15) | | 0.0063 |
| COVID-19 trend (2020) (β3) | -1.78 (-2.7, -0.86) | | 0.0021 |
| DW 0.826, ACF 0.555 | | | |
| **Laboratory follow-up of hypertension** | | | |
| Pre-COVID trend (2011-2019) (β1) | -0.03 (-0.12, 0.07) | | 0.558 |
| COVID-19 impact (March 2020) (β2) | 9.88 (4.73, 15.04) | | 0.0004 |
| COVID-19 trend (2020) (β3) | -1.25 (-3.6, 1.1) | | 0.2542 |
| DW 1.569, ACF 0.197 | | | |
| **Prescribing of medication to paediatric hypertension patients** | | | |
| Pre-COVID trend (2011-2019) (β1) | 0.02 (0.01, 0.02) | | <.0001 |
| COVID-19 impact (March 2020) (β2) | 0.05 (-0.19, 0.29) | | 0.6753 |
| COVID-19 trend (2020) (β3) | -0.04 (-0.14, 0.06) | | 0.3669 |
| DW 1.810, ACF 0.094 | | | |
| **Abbreviations**  DW: Durbin-Watson D  ACF: 1^st^ Order Autocorrelation  BP: blood pressure  95% CI: 95% Confidence Interval | | | |
